# Functional connectome mediates the association between sleep disturbance and mental health in preadolescence: A longitudinal mediation study

**DOI:** 10.1002/hbm.25772

**Published:** 2022-01-18

**Authors:** Fan Nils Yang, Tina Tong Liu, Ze Wang

**Affiliations:** ^1^ Department of Diagnostic Radiology and Nuclear Medicine University of Maryland School of Medicine Baltimore Maryland USA; ^2^ Section on Neurocircuitry, Laboratory of Brain and Cognition National Institute of Mental Health, National Institutes of Health Bethesda Maryland USA

**Keywords:** adolescent psychiatry, cognition, functional neuroimaging, longitudinal mediation analysis, mental health, sleep

## Abstract

Sleep disturbance is known to be associated with various mental disorders and often precedes the onset of mental disorders in youth. Given the increasingly acknowledged bidirectional influence between sleep disturbance and mental disorders, we aim to identify a shared neural mechanism that underlies sleep disturbance and mental disorders in preadolescents. We analyzed a dataset of 9,350 9–10 year‐old children, among whom 8,845 had 1‐year follow‐up data, from the Adolescent Brain Cognitive Development (ABCD) study. Linear mixed‐effects models, mediation analysis, and longitudinal mediation analysis were used to investigate the relationship between sleep disturbance, mental disorders, and resting‐state network connectivity. Out of 186 unique connectivities, the effect of total sleep disturbance (TSP, from Sleep Disturbance Scale) and mental problems (MP, from Child Behavior Checklist) converged in the default mode network (DMN) and the dorsal attention network (DAN). Within‐ and between‐network connectivities (DMN‐DAN, DMN‐DMN, DAN‐DAN) mediated the relationship between baseline TSD and MP at 1‐year follow‐up and the relationship between baseline MP and TSD at 1‐year follow‐up. The pathway model in which sleep disturbance and mental problems affect each other through two anticorrelated brain networks (DMN and DAN) suggests a common neural mechanism between them. Longitudinally, a less segregated DMN and DAN is associated with negative outcomes on mental well‐being and sleep disturbance a year later. These findings have important implications for the design of prevention and neurofeedback intervention for mental disorders and sleep problems.

## Graphical abstract

The impact of sleep disturbance and mental disorders on functional connectivity converges in default mode and dorsal attention networks. The relationship between sleep disturbance and mental disorders is mediated via a shared brain network mechanism. Sleep disturbance and mental disorders at baseline can each predict the other a year later through the identified network connectivities.

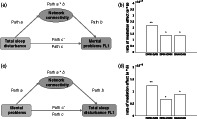

## 1 INTRODUCTION

Many mental disorders share similar risk factors and/or brain alterations, and respond to the same therapies (Caspi & Moffitt, 2018). Both theoretical and empirical work has suggested that this comorbidity might be due to the nonspecificity of the functional neurocircuit (Lees et al., 2021; McTeague et al., 2017; Menon, 2011; Sha, Wager, Mechelli, & He, 2019). Specifically, according to the triple network model, a wide variety of psychopathologies are associated with aberrant functional connectivity within and between three large‐scale neurocognitive networks, that is, salience network, frontoparietal network, and default mode network, as well as subnetworks such as ventral and dorsal attention networks (Menon, 2011). This conceptual model has been corroborated by many recent studies (Lees et al., 2021; McTeague et al., 2017; Sha et al., 2019; Xia et al., 2018), including those showing that common functional network disruptions in abovementioned networks were linked to all dimensions of psychopathology in preadolescents (Lees et al., 2021) and adolescents (Xia et al., 2018).

Moreover, individuals with mental disorders often experience varying degrees of sleep disturbance (Baglioni et al., 2016; Tesler, Gerstenberg, & Huber, 2013). Recent studies showed that sleep disturbance and mental disorders could aggravate each other in a reciprocal manner (Alfano, Ginsburg, & Kingery, 2007; Cox & Olatunji, 2016; Gregory & Sadeh, 2016; Hansen, Skirbekk, Oerbeck, Wentzel‐Larsen, & Kristensen, 2014; Tesler et al., 2013). On the one hand, longitudinal studies have shown that sleep disturbance in youth likely precedes and exacerbates symptoms of attention deficit hyperactivity disorder (ADHD) (Scott et al., 2013) and mood and anxiety disorders (Goldstone et al., 2020; Gregory et al., 2005; Gregory, Rijsdijk, Lau, Dahl, & Eley, 2009; Jansen et al., 2011). On the other hand, greater polygenic risk factors for various mental disorders, that is, ADHD, mood and anxiety disorders, may contribute to greater sleep disturbance among children (Ohi et al., 2021). These findings strongly suggest a potential shared neural mechanism between sleep disturbance and mental disorders, which has not been demonstrated to date.

The purpose of this study was to address this open question using resting‐state functional connectivity (rs‐FC), a widely‐used fMRI technique for studying brain networks and cognition as well as their alterations in brain diseases (Gabrieli, Ghosh, & Whitfield‐Gabrieli, 2015; Woo, Chang, Lindquist, & Wager, 2017). rs‐FC measure is highly reproducible within an individual across scan sessions (Finn et al., 2015; Noble, Scheinost, & Constable, 2019). Thus, it has been recognized as a promising neural biomarker for assessing neurocognitive development and identifying aspects of intrinsic brain network organization that are related to cognition and disease status. In particular, altered functional connectivity within and between the default mode network and its anticorrelated networks, including dorsal and ventral attention networks, were found in sleep‐deprived adults (Chee & Zhou, 2019; De Havas, Parimal, Soon, & Chee, 2012; Kaufmann et al., 2016; Sämann et al., 2010).

So far, very few studies have investigated the associations between functional connectivity and sleep disturbance in preadolescence, which is a critical period for brain development. Coincidentally, the onset of mental disorders often starts around the same time, that is, childhood or adolescence (Kessler et al., 2007; Tesler et al., 2013). To fill this gap of knowledge and to understand the relationship between sleep disturbance, mental disorders, and functional connectivity in preadolescence, we analyzed the large dataset from the Adolescent Brain Cognitive Development (ABCD, https://abcdstudy.org) study, the largest observational and normative project (over 11,000 children) on brain development and child health to date. The nature of the large ABCD dataset offers a unique opportunity to reveal relatively small effects of sleep and mental health on functional brain networks, which cannot be reliably detected under small sample size.

The main goal of the current study is to identify a shared network mechanism between sleep disturbance and mental disorders, if there is any, and to test whether the identified network connectivity mediates the association between mental disorders and sleep disturbance. Out of 186 unique connectivity measures tested, the effect of sleep disturbance and mental disorders converged in the between‐ and within‐network connectivities in the default mode network (DMN) and one of its anticorrelated networks (i.e., dorsal attention network, DAN). The same network connectivities mediated the association between sleep disturbance and mental disorders. Additional longitudinal analyses demonstrated that the three network connectivities mediated the effect of sleep disturbance on mental disorders 1 year later and the effect of mental disorders on sleep disturbance 1 year later.

## 2 MATERIALS AND METHODS

### 2.1 Data source

The ABCD study (data release 2.01 used in the current study) includes baseline data from more than 11,000 9–10‐year‐old (Casey et al., 2018). Data were collected from 21 research sites across the United States, approved by institutional review boards (IRB) at the University of California, San Diego as well as at each local site. Parents' written informed consent and children's assent were obtained at each site. Recruitment followed demographic distribution (sex, race, ethnicity, household income, etc.) of the general population in the United States. Children with serious neurological or psychiatric diagnoses were excluded. Details about the protocols are available at the ABCD study website (https://abcdstudy.org/scientists/protocols/).

In total, data from 11,878 children are provided at baseline. Among them, 9,387 passed the rsfmri QC (imgincl_rsfmri_include) provided by the ABCD study (Hagler et al., 2019). An additional 37 children were excluded due to missing values on network connectivity (*n* = 14), mental problems (*n* = 5), or total sleep disturbance (*n* = 21) (three of them have more than one missing values). Thus, the total number of children included in the current study was 9,350. Among whom 8,845 had 1‐year follow‐up data. Table 1 provides detailed demographic information.

**TABLE 1:** Demographic information of the participants from the ABCD dataset included in the present study

Characteristic	Baseline	One‐year follow‐up
	*N* (%) or mean (SD)	*N* (%) or mean (SD)
Age (months)	119.3 (7.5)	131.4 (7.8)
Sex at birth		
Female	4,674 (50.0%)	4,412 (49.9%)
Male	4,676 (50.0%)	4,433 (50.1%)
Race		
White	6,101 (65.3%)	5,872 (66.4%)
Black	1,340 (14.3%)	1,198 (13.5%)
Other (mixes or Asians)	1909 (20.4%)	1775 (20.1%)
Pubertal status	1.8 (0.9)^a^	2.1 (1.0)^b^
Mental problems	45.4 (11.2)	45.0 (11.0)^c^
MP ≥ 67 (clinical threshold)	2,468 (26.4%)	2,166 (24.5%)
Total sleep disturbance	36.3 (8.0)	36.5 (7.9)^d^
TSD ≥ 52 (clinical threshold)	463 (5.0%)	474 (5.4%)
Total cognitive composite score	48.3 (11.1)^e^	N/A^f^
Number of children	9,350	8,845

Abbreviations: MP, mental problems; TSD, total sleep disturbance.

^a^ Three hundred and five children have missing values.

^b^ Three thousand and ninety children have missing values.

^c^ Thirty‐three children have missing values.

^d^ Twenty‐eight children have missing values.

^e^ Seven hundred and fifty‐two children have missing values.

^f^ Total cognitive composite score did not have 1‐year follow‐up data.

### 2.2 Sleep measures

Total sleep disturbance (TSD) and its 1‐year follow‐up was calculated from the ABCD Parent‐reported Sleep Disturbance Scale for Children (abcd_sdss01) as the sum of six scores corresponding to six different sleep disorders, including disorders of initiating and maintaining sleep, sleep breathing disorders, disorder of arousal, sleep–wake transition disorders, disorders of excessive somnolence, and sleep hyperhidrosis (Bruni et al., 1996).

### 2.3 Mental health measures

Children's dimensional psychopathology and adaptive functioning were assessed by the Parent‐reported Child Behavior Checklist Scores (abcd_cbcls01). The total score of psychiatric problems (total problems *t* score, as an index of mental problems, MP) and its 1‐year follow‐up was calculated based on scores of 8 empirically‐based syndromes, including anxious/depressed, withdrawn/depressed, somatic complaints, social problems, thought problems, attention problems, rule‐breaking behavior, and aggressive behavior (Achenbach & Rescorla, 2004).

### 2.4 Cognitive measures

Total Cognitive Composite Fully‐Corrected T‐score (abcd_tbss01, baseline only, no data available at 1‐year follow‐up) of the NIH Cognition Battery Toolbox was used as the measure for general cognitive function (Akshoomoff et al., 2013). It is the sum of the scores from seven cognitive components: language vocabulary knowledge, attention, cognitive control, working memory, executive function, episodic memory, and language.

### 2.5 Network connectivity

Participants completed four runs of 5‐min resting‐state fMRI scan (20 min in total) while instructed to look at a fixation crosshair. Preprocessing of the functional connectivity analysis and the network connectivity strengths were provided by the ABCD consortium. Detailed MRI scan parameters and preprocessing steps have been previously reported and discussed (Hagler et al., 2019). Briefly, preprocessing steps of rs‐FC included registration, distortion correction, normalization, regression of covariates (24 motion parameters, outliers with framewise displacement higher than 3 mm, and signals from white matter, cerebral spinal fluid, and whole‐brain). Time points with framewise‐displacement (FD) higher than 0.2 mm were excluded from the connectivity calculation. Within‐ or between‐network connectivities were calculated as the average fisher‐transferred functional connectivity between each pair of ROIs within or between networks (12 networks in total) based on the Gordon atlas (Gordon et al., 2016). In addition, network connectivities between each network and each of the nine subcortical regions, including cerebellum cortex, thalamus, caudate, putamen, pallidum, hippocampus, amygdala, accumbens area, ventral Diencephalon (ventraldc), were also calculated based on the Fischl et al. (2002) atlas. Quality controls were performed by trained ABCD staff (Hagler et al., 2019) and were used as an inclusion criterion. Sites effects were harmonized by the ComBat method (Johnson, Li, & Rabinovic, 2007; Yang et al., 2021; Yu et al., 2018), which can successfully remove site effects on brain connectivities and increase the power to detect effects of interest. Network connectivity measures were not available at 1‐year follow‐up.

### 2.6 Statistical analyses

We first tested whether TSD and MP were correlated. Then, Linear Mixed‐effects Models (LME, implemented through function *fitlme* in Matlab) were used to investigate the effect of MP or TSD on network connectivity. All models included fixed‐effect covariates for age, sex at birth, race (black, white, and others), pubertal status (1–4, assessed by ABCD Youth Pubertal Development Scale and Menstrual Cycle Survey History), average motion during resting scan (mean FD), and random effects for family relatives nested within data collection sites. As the distribution of TSD was skewed, we log‐transformed the data to approximate normality (the results were the same using non‐log‐transformed data). We performed additional sensitivity analyses to ensure our results are robust even after including a wide‐range of covariates, that is, number of fMRI time points remained after preprocessing, household income, parents' education, body mass index (see Figures S3 and S4). Comparable results were obtained with and without including these additional covariates. False discovery rate (FDR, alpha set to 0.05, 186 unique comparisons in total) was applied to correct for multiple comparisons. The relationships between cognition, identified network connectivity, total sleep disturbance and mental problems were investigated, results were reported in the Supporting Information (see Figure S2).

The mediation toolbox (https://github.com/canlab/MediationToolbox) was used to perform all the mediation analyses (Wager, Davidson, Hughes, Lindquist, & Ochsner, 2008; Wager et al., 2009). Here, two sets of mediation models were performed. For the first set (see Figure 2a), the independent variable (*X*) was TSD, the dependent variable (*Y*) was MP, and the mediator (*M*) was the network connectivities identified through LME. For the second set (see Figure 2c), the independent variable was MP, the dependent variable was TSD, and the mediator was the network connectivities identified through LME. The test of mediation involves two linear equations (see Equations 1 and 2). The path coefficient *a* reflects the effects of *X* on *M*. The path coefficient *b* effect reflects the effect of *M* on *Y*. The coefficient *c*′ is the direct effect of *X* on *Y* after controlling for *M*. The product *a* * *b* (mediation effect/indirect effect) reflects how the association between *X* and *Y* changed according to *M*. *d*_1_ and *d*_2_ are intercept terms (content). The total effect *c* (not shown in the equation, *c* = *a* * *b* + *c*′) is the effect of *X* on *Y* without controlling for *M*. All abovementioned covariates (age, sex, race, pubertal status, family relatives, and data collection sites) were controlled in the mediation analyses. The significance of the mediation analyses was estimated using bootstrap sampling with 10,000 random‐generated samples on the product of the *a and b* path coefficients (*a* * *b*).

(1) $$M=\mathrm{aX}+d_{1},$$

(2) $$Y=\mathrm{bM}+c^{\prime }X+d_{2}.$$

### 2.7 Longitudinal mediation analyses

Eight thousand eight hundred and forty‐five of the 9,350 children participated in the 1‐year follow‐up study (see Table 1). Mediation analyses were performed to test the longitudinal associations between TSD, MP, and network connectivities. Specifically, we tested whether identified network connectivities mediated the associations between TSD at baseline and MP 1 year later (controlled for baseline MP), and between MP at baseline and TSD 1 year later (controlled for baseline TSD). Additional covariates including baseline age, sex, race, baseline pubertal status, family relatives, and sites. The significance of the longitudinal mediation analyses was estimated using bootstrap sampling with 10,000 random‐generated samples.

## 3 RESULTS

Out of the 11,878 children, 9,350 were included in the current analysis after removing participants with missing behavioral data and imaging data that failed quality control. Eight thousand eight hundred and forty‐five of the 9,350 children had 1‐year follow‐up data. Table 1 summarizes the demographic variables of baseline and follow‐up data. As expected, in the current study, TSD and MP were significantly correlated (*r* = .568, *p* < 1e−10, see Figure S1), after controlling for age, sex, race, pubertal status, effects for family relatives, mean FD (the relative motions between adjacent image volumes), and data collection sites. Additional analysis indicated that sex did not significantly change the relationship between TSD and MP (see Supporting Information).

### 3.1 Total sleep disturbance and network connectivity

**FIGURE 1:**
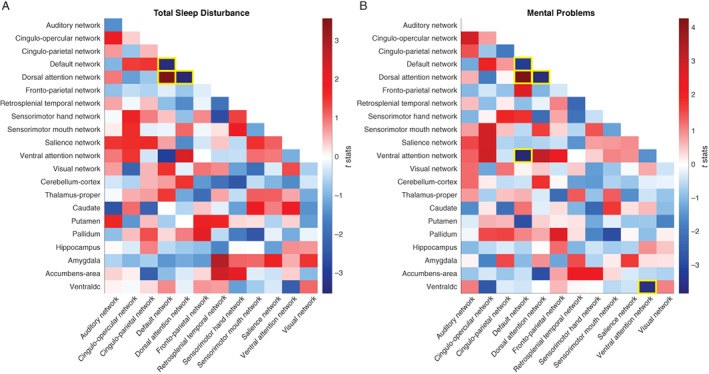
The associations between network connectivity measures and total sleep disturbance/mental problems. (a) The association between network connectivities and total sleep disturbance. Color bar represents *t*‐stats. That is, red/blue means positive/negative association between total sleep disturbance and network connectivity measures, respectively. Only unique network connectivity measures were shown in the matrix (i.e., top right was intentionally left blank). Yellow outlines denote network connectivity that survived FDR correction (*p* < .05 FDR corrected) for multiple comparisons out of 186 unique comparisons. (b) The association between network connectivities and mental problems. Color bar represents *t*‐stats. That is, red/blue means positive/negative association between mental problems and network connectivity measures, respectively. Yellow outlines denote network connectivity that survived FDR correction (*p* < .05 FDR corrected) for multiple comparisons out of 186 unique comparisons. Ventraldc, ventral Diencephalon

The distribution of TSD was skewed, so we log‐transformed the data to approximate normality. In total, 78 within‐ and between‐network connectivities and 108 network‐subcortical connectivities were tested (Figure 1a). Out of the 186 unique comparisons, TSD had a significant impact on only three network connectivities with FDR correction (see yellow outlines in Figure 1a), DMN‐DAN, DMN‐DMN, and DAN‐DAN. Comparable results were found when four additional covariates were added, see Figure S3.

### 3.2 Mental problems and network connectivity

Similarly, the effect of MP on 186 unique network connectivities were tested. Five associations between MP and network connectivity survived FDR correction (see yellow outlines in Figure 1b). Interestingly, the same three network connectivities impacted by TSD were also influenced by MP, that is, DMN‐DAN, DMN‐DMN, and DAN‐DAN. Comparable results were found when four additional covariates were added, see Figure S4.

### 3.3 Mediation analysis

**FIGURE 2:**
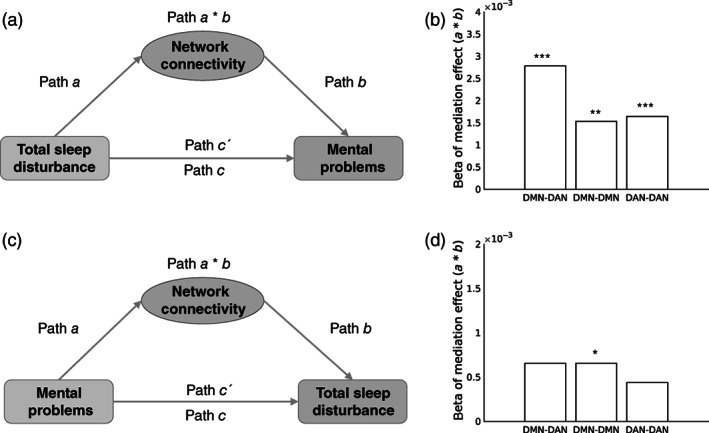
Network connectivities mediated the relationship between total sleep disturbance and mental problems (a and b) and the relationship between mental problems and total sleep disturbance (c and d). (a) Diagram of the first set of mediation models. These models test whether identified network connectivities (DMN‐DAN, DMN‐DMN, and DAN‐DAN) mediated the effect of TSD on MP (see Section 2 for details). (b) Bar graph of mediation effects of the three network connectivities in the first set of mediation models. All beta of path *a* * *b* was significant (all *p* < .005). (c) Diagram of the second set of mediation models, these models test whether identified network connectivities (DMN‐DAN, DMN‐DMN, and DAN‐DAN) mediated the effect of MP on TSD (see Section 2 for details). (d) Bar graph of mediation effects of the three network connectivities in the second set of mediation models. Only DMN‐DMN mediated the effect of MP on TSD (*p* < .05). *p < .05; **p < .01; ***p < .001. DAN, dorsal attention network; DMN, default mode network; MP, mental problems; TSD, total sleep disturbance

Given that the impact of TSD and MP converged on the same three network connectivities (DMN‐DAN, DMN‐DMN, DAN‐DAN), we further conducted a mediation analysis to explore the underlying mechanism by which TSD and MP influence each other. In other words, we tested whether these three network connectivities (DMN‐DAN, DMN‐DMN, or DAN‐DAN) mediated the relationship between total sleep disturbance and mental problems. We found that all three network connectivity measurements significantly mediated the effect of TSD on MP (all *p* < .005; 95% CI did not include 0, see Figure 2b). However, only the within‐network connectivity (DMN‐DMN) significantly mediated the effect of MP on TSD (*p* = .015; *β* = .0007; 95% CI, 0.0001–0.0014, see Figure 2d).

### 3.4 Longitudinal mediation analysis

**FIGURE 3:**
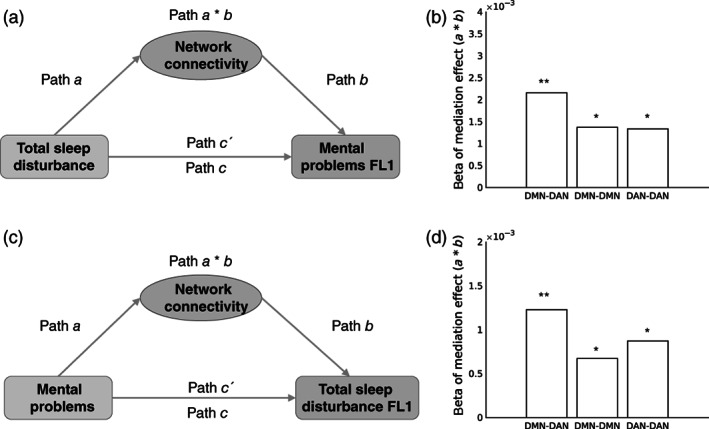
Network connectivities mediated both the relationship between total sleep disturbance (at baseline) and mental problems (at 1‐year follow‐up, FL1) (a and b) and the relationship between mental problems (at baseline) and total sleep disturbance (at FL1) (c and d). (a) Diagram of the first set of mediation models. These models test whether identified network connectivities (DMN‐DAN, DMN‐DMN, and DAN‐DAN) mediated the effect of TSD on MP at FL1. (b) Bar graph of mediation effects of the three network connectivities in the first set of mediation models. All beta of path *a* * *b* was significant (all *p* < .05). (c) Diagram of the second set of mediation models, these models test whether identified network connectivities (DMN‐DAN, DMN‐DMN, and DAN‐DAN) mediated the effect of MP on TSD at FL1. (d) Bar graph of mediation effects of the three network connectivities in the second set of mediation models. All beta of path *a* * *b* was significant (all *p* < .05). Each longitudinal mediation analysis controlled for the corresponding baseline data of the dependent variable, for example, for diagram A, baseline mental problems was added as a covariate. *p < .05; **p < .01. DAN, dorsal attention network; DMN, default mode network; MP, mental problems; TSD, total sleep disturbance

Longitudinal mediation analysis revealed that all three network connectivities (DMN‐DAN, DMN‐DMN, and DAN‐DAN) mediated the effect of TSD at baseline on MP 1 year later, after controlling for baseline MP (all *p* < .05; 95% CI did not include 0, see Figure 3b). Interestingly, distinct from the pattern that only DMN‐DMN mediated the effect of MP on TSD at baseline, all three network connectivities mediated the effect of baseline MP on TSD at 1 year later, after adjusting for baseline TSD (all *p* < .05; 95% CI did not include 0, see Figure 3d).

## 4 DISCUSSION

Based on data from a large cohort of preadolescents enrolled in the ABCD study, our work identified robust relationships between sleep disturbance, mental health, and functional connectivity of many brain networks. Total sleep disturbance and mental problems both impacted similar network connectivities and their relationship was mediated by these identified network connectivities. The effect of sleep disturbance on mental problems was mediated by three network connectivities, that is, DMN‐DAN, DMN‐DMN, and DAN‐DAN. In contrast, only DMN‐DMN mediated the effect of mental problems on sleep disturbance. Follow‐up analyses showed that the anticorrelation between DMN and DAN was significantly associated with the total cognition score. Longitudinal analysis revealed that total sleep disturbance and mental problems at baseline can each predict the other 1 year later through these three network connectivities (i.e., DMN‐DAN, DMN‐DMN, and DAN‐DAN). These results supported our hypothesis that sleep disturbance and mental disorders have a similar impact on rs‐FC. Using a data‐driven approach, we discovered that their impact converged in the between‐ and within‐network connectivities in the DMN and one of its anticorrelated networks, DAN. Our findings suggest a common neural mechanism through which sleep disturbance and mental problems may exacerbate each other.

DMN is generally considered an integrated system that is associated with many different aspects of self‐related mental processes such as autobiographical memory, internal thoughts, emotion regulations (Menon, 2011). Given these roles, it is not surprising that abnormal connectivity within DMN has been involved in almost every major psychiatric disorder, including dementia, schizophrenia, anxiety and depression, autism, and ADHD (Broyd et al., 2009). Consistent with these observations, we found that mental problems were negatively correlated with DMN's within‐network connectivity (DMN‐DMN) in the current study. That is, higher scores in mental problems correspond to weaker connectivities between brain regions within DMN. Moreover, we demonstrated that mental problems had an indirect detrimental effect on sleep disturbance through DMN‐DMN in preadolescence.

Unlike DMN, DAN is considered a “task‐positive” network (Fox et al., 2005). Under externally directed cognitive tasks (e.g., visual search), DAN activation and DMN deactivation usually co‐occur. This anticorrelation between DMN and DAN emerges in childhood and continues to develop during adolescence (Fair et al., 2009). In the current study, we found that both sleep disturbance and mental problems were associated with the connectivity strength between DMN and DAN. Moreover, we found that the degree to which DMN and DAN are segregated as two anticorrelated networks was negatively correlated with the total cognition scores, suggesting that the segregation between DMN and DAN might be a critical factor accounting for one's cognitive performance. These results are in line with a previous study showing that maturation of network modules, including segregation of DMN and its anticorrelated networks, during neurodevelopment can be a critical driver of cognitive development (Gu et al., 2015).

Emerging evidence suggests that symptom severity of various mental disorders and sleep disturbance could impact each other in a bidirectional manner (Tesler et al., 2013). For example, less sleep disturbance was found in children who received treatment for anxiety disorders compared to those who received placebo (Alfano et al., 2007). In youth with mood disorders, sleep disturbance was associated with more severe symptomatology, longer episodes, and increased risk for relapse (Emslie et al., 2012; Liu et al., 2007). To the best of our knowledge, the current study first explains the co‐occurrence and bidirectionality of mental disorders and sleep disturbance by identifying a shared network mechanism between sleep disturbance and mental problems, that is, within‐ and between‐network connectivity in DMN and DAN. Mediation analysis and longitudinal mediation analysis further confirmed that sleep disturbance and mental disorders could affect each other via these three network connectivities (DMN‐DAN, DAN‐DAN, and DMN‐DMN). Finally, we note another non‐mutually exclusive possibility of the relationship between MP and TSD. In addition to a bidirectional influence between MP and TSD, they could also be seen as highly comorbid problems, or two aspects of a single condition.

The specificity of the functional networks (DMN‐DAN, DAN‐DAN, and DMN‐DMN) as the mediator identified throughout longitudinal mediation analyses may guide future neuromodulation studies by targeting these network functional connectivities. For example, real‐time fMRI neurofeedback is an emerging noninvasive technique for modulating aberrant neurocircuitry, with the potential to induce long‐term symptom reductions in patients with various mental disorders (Linhartová et al., 2019). In our pathway model, the DMN‐DAN connectivity mediated both the bidirectional and longitudinal relationship between mental disorders and sleep disturbance. Thus, regulating DMN‐DAN connectivity strength can potentially lead to reduced mental problems and sleep disturbance, and potentially yielded a positive and long‐term effect in individuals at‐risk for mental disorders and sleep disturbance.

Several limitations in the current study should be noted. First, the effect sizes found in the current study with a large sample size are relatively small compared to those reported in the literature based on much smaller sample sizes. Despite a small *r*‐value of the correlation between DMN‐DAN and total cognition (−.036), according to ABCD analysis guidelines (Dick et al., 2021), a −.036 *r* value in about 10,000 participants has statistical power higher than .9, which is considered well‐powered. In addition, a previous large sample‐size study found even the most significant results can only explain around 1% of the total variances (Miller et al., 2016). Second, we focused on the high‐level commonality between sleep disturbance and various mental disorders (rather than each type of mental disorder). Thus, we did not investigate the relationship between sleep disturbance and each specific type of mental disorder. Caveat is needed when interpreting the current findings with regard to specific mental disorders, for example, depression, anxiety disorders, and so forth. Third, the associations between mental problems and network connectivity established in the current study were found in a comparable fashion in a previous study (Lees et al., 2021). Specifically, Lees et al. (2021) reported that all dimensions of general psychopathology were associated with higher within‐network connectivity within both DAN and retrosplenial‐temporal network. In contrast, lower between‐network connectivities between the ventral attention and frontoparietal regions, and between DAN and the amygdala were also reported. Potential sources for this discrepancy could be a combination of (a) two sets of mental problem index used (i.e., all dimensions of general psychopathology generated from Affective Disorders and Schizophrenia for School‐Age Children for DSM‐5 was used in Lees et al. (2021), whereas mental problems from Child Behavior Checklist Scores was used in the current study, and (b) a different number of covariates included (i.e., more covariates are used in the current study, such as age, mean FD, pubertal status.

## 5 CONCLUSION

Taken together, our results shed light on a shared and stable neural mechanism between sleep disturbance and mental disorders. The network connectivity between DMN and DAN that mediated the bidirectional and long‐term relationship between sleep disturbance and mental disorders could be a promising key target for neurofeedback training or behavioral therapy. Future work may examine the effectiveness of regulating connectivity strength between DMN and DAN in inducing positive behavioral change related to mental health and sleep problems.

## CONFLICT OF INTEREST

The authors declare no competing interests.

## AUTHOR CONTRIBUTIONS

Fan Nils Yang conceptualized the study, analyzed the data, generated figures and wrote the original draft. Tina Tong Liu contributed to conceptualization and visualization, and edited the manuscript. Ze Wang conceptualized the initial study, supervised the study and interpretation, and edited the manuscript.

## ETHICS STATEMENT

ABCD study received ethical approval in accordance with the ethical standards of the 1964 Declaration of Helsinki.

## CODE AVAILABILITY

Custom code used in this manuscript can be obtained upon request from the authors.

## Supporting information


**Appendix** **S1**: Supporting information.

## Data Availability

The ABCD data that are used by this study are available in National Institutes of Mental Health Data Archive (NDA): https://nda.nih.gov/abcd.

## References

[hbm25772-bib-0001] Achenbach, T. M. , & Rescorla, L. A. (2004). The Achenbach system of empirically based assessment (ASEBA) for ages 1.5 to 18 years. In The use of psychological testing for treatment planning and outcomes assessment: Instruments for children and adolescents (Vol. 2, 3rd ed., pp. 179–213). Mahwah, NJ: Lawrence Erlbaum Associates Publishers.

[hbm25772-bib-0002] Akshoomoff, N. , Beaumont, J. L. , Bauer, P. J. , Dikmen, S. S. , Gershon, R. C. , Mungas, D. , … Zelazo, P. D. (2013). VIII. NIH toolbox cognition battery (CB): Composite scores of crystallized, fluid, and overall cognition. Monographs of the Society for Research in Child Development, 78, 119–132. 23952206 10.1111/mono.12038 PMC4103789

[hbm25772-bib-0003] Alfano, C. A. , Ginsburg, G. S. , & Kingery, J. N. (2007). Sleep‐related problems among children and adolescents with anxiety disorders. Journal of the American Academy of Child and Adolescent Psychiatry, 46, 224–232. 10.1097/01.chi.0000242233.06011.8e 17242626

[hbm25772-bib-0004] Baglioni, C. , Nanovska, S. , Regen, W. , Spiegelhalder, K. , Feige, B. , Nissen, C. , … Riemann, D. (2016). Sleep and mental disorders: A meta‐analysis of polysomnographic research. Psychological Bulletin, 142, 969–990. 10.1037/bul0000053 27416139 PMC5110386

[hbm25772-bib-0005] Broyd, S. J. , Demanuele, C. , Debener, S. , Helps, S. K. , James, C. J. , & Sonuga‐Barke, E. J. S. (2009). Default‐mode brain dysfunction in mental disorders: A systematic review. Neuroscience and Biobehavioral Reviews, 33, 279–296. 10.1016/j.neubiorev.2008.09.002 18824195

[hbm25772-bib-0006] Bruni, O. , Ottaviano, S. , Guidetti, V. , Romoli, M. , Innocenzi, M. , Cortesi, F. , & Giannotti, F. (1996). The Sleep Disturbance Scale for children (SDSC). Construction and validation of an instrument to evaluate sleep disturbances in childhood and adolescence. Journal of Sleep Research, 5, 251–261. 10.1111/j.1365-2869.1996.00251.x 9065877

[hbm25772-bib-0007] Casey, B. J. , Cannonier, T. , Conley, M. I. , Cohen, A. O. , Barch, D. M. , Heitzeg, M. M. , … Imaging Acquisition Workgroup, A. B. C. D. (2018). The adolescent brain cognitive development (ABCD) study: Imaging acquisition across 21 sites. Developmental Cognitive Neuroscience, 32, 43–54. 10.1016/j.dcn.2018.03.001 29567376 PMC5999559

[hbm25772-bib-0008] Caspi, A. , & Moffitt, T. E. (2018). All for one and one for all: Mental disorders in one dimension. The American Journal of Psychiatry, 175, 831–844. 10.1176/appi.ajp.2018.17121383 29621902 PMC6120790

[hbm25772-bib-0009] Chee, M. W. L. , & Zhou, J. (2019). Functional connectivity and the sleep‐deprived brain. In Progress in brain research (pp. 159–176). Cambridge, MA: Elsevier. 10.1016/bs.pbr.2019.02.009 31072560

[hbm25772-bib-0010] Cox, R. C. , & Olatunji, B. O. (2016). A systematic review of sleep disturbance in anxiety and related disorders. Journal of Anxiety Disorders, 37, 104–129. 10.1016/j.janxdis.2015.12.001 26745517

[hbm25772-bib-0011] De Havas, J. A. , Parimal, S. , Soon, C. S. , & Chee, M. W. L. (2012). Sleep deprivation reduces default mode network connectivity and anti‐correlation during rest and task performance. NeuroImage, 59, 1745–1751. 10.1016/j.neuroimage.2011.08.026 21872664

[hbm25772-bib-0012] Dick, A. S. , Lopez, D. A. , Watts, A. L. , Heeringa, S. , Reuter, C. , Bartsch, H. , … Thompson, W. K. (2021). Meaningful associations in the adolescent brain cognitive development study. NeuroImage, 239, 118262. 10.1016/j.neuroimage.2021.118262 34147629 PMC8803401

[hbm25772-bib-0013] Emslie, G. J. , Kennard, B. D. , Mayes, T. L. , Nakonezny, P. A. , Zhu, L. , Tao, R. , … Croarkin, P. (2012). Insomnia moderates outcome of serotonin‐selective reuptake inhibitor treatment in depressed youth. Journal of Child and Adolescent Psychopharmacology, 22, 21–28. 10.1089/cap.2011.0096 22257126 PMC3281293

[hbm25772-bib-0014] Fair, D. A. , Cohen, A. L. , Power, J. D. , Dosenbach, N. U. F. , Church, J. A. , Miezin, F. M. , … Petersen, S. E. (2009). Functional brain networks develop from a “local to distributed” organization. PLoS Computational Biology, 5, e1000381. 10.1371/journal.pcbi.1000381 19412534 PMC2671306

[hbm25772-bib-0015] Finn, E. S. , Shen, X. , Scheinost, D. , Rosenberg, M. D. , Huang, J. , Chun, M. M. , … Constable, R. T. (2015). Functional connectome fingerprinting: Identifying individuals using patterns of brain connectivity. Nature Neuroscience, 18, 1664–1671. 10.1038/nn.4135 26457551 PMC5008686

[hbm25772-bib-0016] Fischl, B. , Salat, D. H. , Busa, E. , Albert, M. , Dieterich, M. , Haselgrove, C. , … Dale, A. M. (2002). Whole brain segmentation: Automated labeling of neuroanatomical structures in the human brain. Neuron, 33, 341–355. 10.1016/s0896-6273(02)00569-x 11832223

[hbm25772-bib-0017] Fox, M. D. , Snyder, A. Z. , Vincent, J. L. , Corbetta, M. , Van Essen, D. C. , & Raichle, M. E. (2005). The human brain is intrinsically organized into dynamic, anticorrelated functional networks. Proceedings of the National Academy of Sciences, 102, 9673–9678. 10.1073/pnas.0504136102 PMC1157105 15976020

[hbm25772-bib-0018] Gabrieli, J. D. E. , Ghosh, S. S. , & Whitfield‐Gabrieli, S. (2015). Prediction as a humanitarian and pragmatic contribution from human cognitive neuroscience. Neuron, 85, 11–26. 10.1016/j.neuron.2014.10.047 25569345 PMC4287988

[hbm25772-bib-0019] Goldstone, A. , Javitz, H. S. , Claudatos, S. A. , Buysse, D. J. , Hasler, B. P. , de Zambotti, M. , … Baker, F. C. (2020). Sleep disturbance predicts depression symptoms in early adolescence: Initial findings from the adolescent brain cognitive development study. The Journal of Adolescent Health, 66, 567–574. 10.1016/j.jadohealth.2019.12.005 32046896 PMC7183901

[hbm25772-bib-0020] Gordon, E. M. , Laumann, T. O. , Adeyemo, B. , Huckins, J. F. , Kelley, W. M. , & Petersen, S. E. (2016). Generation and evaluation of a cortical area Parcellation from resting‐state correlations. Cerebral Cortex, 26, 288–303. 10.1093/cercor/bhu239 25316338 PMC4677978

[hbm25772-bib-0021] Gregory, A. M. , Caspi, A. , Eley, T. C. , Moffitt, T. E. , Oconnor, T. G. , & Poulton, R. (2005). Prospective longitudinal associations between persistent sleep problems in childhood and anxiety and depression disorders in adulthood. Journal of Abnormal Child Psychology, 33, 157–163. 10.1007/s10802-005-1824-0 15839494

[hbm25772-bib-0022] Gregory, A. M. , Rijsdijk, F. V. , Lau, J. Y. F. , Dahl, R. E. , & Eley, T. C. (2009). The direction of longitudinal associations between sleep problems and depression symptoms: A study of twins aged 8 and 10 years. Sleep, 32, 189–199. 19238806 10.1093/sleep/32.2.189 PMC2635583

[hbm25772-bib-0023] Gregory, A. M. , & Sadeh, A. (2016). Annual research review: Sleep problems in childhood psychiatric disorders—A review of the latest science. Journal of Child Psychology and Psychiatry, 57, 296–317. 10.1111/jcpp.12469 26412255

[hbm25772-bib-0024] Gu, S. , Satterthwaite, T. D. , Medaglia, J. D. , Yang, M. , Gur, R. E. , Gur, R. C. , & Bassett, D. S. (2015). Emergence of system roles in normative neurodevelopment. Proceedings of the National Academy of Sciences, 112, 13681–13686. 10.1073/pnas.1502829112 PMC4640772 26483477

[hbm25772-bib-0025] Hagler, D. J. , Hatton, S. N. , Cornejo, M. D. , Makowski, C. , Fair, D. A. , Dick, A. S. , … Dale, A. M. (2019). Image processing and analysis methods for the adolescent brain cognitive development study. NeuroImage, 202, 116091. 10.1016/j.neuroimage.2019.116091 31415884 PMC6981278

[hbm25772-bib-0026] Hansen, B. H. , Skirbekk, B. , Oerbeck, B. , Wentzel‐Larsen, T. , & Kristensen, H. (2014). Associations between sleep problems and attentional and behavioral functioning in children with anxiety disorders and ADHD. Behavioral Sleep Medicine, 12, 53–68. 10.1080/15402002.2013.764525 23461477

[hbm25772-bib-0027] Jansen, P. W. , Saridjan, N. S. , Hofman, A. , Jaddoe, V. W. V. , Verhulst, F. C. , & Tiemeier, H. (2011). Does disturbed sleeping precede symptoms of anxiety or depression in toddlers? The generation R study. Psychosomatic Medicine, 73, 242–249. 10.1097/PSY.0b013e31820a4abb 21257976

[hbm25772-bib-0028] Johnson, W. E. , Li, C. , & Rabinovic, A. (2007). Adjusting batch effects in microarray expression data using empirical Bayes methods. Biostatistics, 8, 118–127. 10.1093/biostatistics/kxj037 16632515

[hbm25772-bib-0029] Kaufmann, T. , Elvsåshagen, T. , Alnæs, D. , Zak, N. , Pedersen, P. Ø. , Norbom, L. B. , … Westlye, L. T. (2016). The brain functional connectome is robustly altered by lack of sleep. NeuroImage, 127, 324–332. 10.1016/j.neuroimage.2015.12.028 26712339 PMC6600874

[hbm25772-bib-0030] Kessler, R. C. , Amminger, G. P. , Aguilar‐Gaxiola, S. , Alonso, J. , Lee, S. , & Ustün, T. B. (2007). Age of onset of mental disorders: A review of recent literature. Current Opinion in Psychiatry, 20, 359–364. 10.1097/YCO.0b013e32816ebc8c 17551351 PMC1925038

[hbm25772-bib-0031] Lees, B. , Squeglia, L. M. , McTeague, L. M. , Forbes, M. K. , Krueger, R. F. , Sunderland, M. , … Mewton, L. (2021). Altered neurocognitive functional connectivity and activation patterns underlie psychopathology in preadolescence. Biological Psychiatry: Cognitive Neuroscience and Neuroimaging, 6, 387–398. 10.1016/j.bpsc.2020.09.007 33281105 PMC8426459

[hbm25772-bib-0032] Linhartová, P. , Látalová, A. , Kóša, B. , Kašpárek, T. , Schmahl, C. , & Paret, C. (2019). fMRI neurofeedback in emotion regulation: A literature review. NeuroImage, 193, 75–92. 10.1016/j.neuroimage.2019.03.011 30862532

[hbm25772-bib-0033] Liu, X. , Buysse, D. J. , Gentzler, A. L. , Kiss, E. , Mayer, L. , Kapornai, K. , … Kovacs, M. (2007). Insomnia and hypersomnia associated with depressive phenomenology and comorbidity in childhood depression. Sleep, 30, 83–90. 17310868 10.1093/sleep/30.1.83

[hbm25772-bib-0034] McTeague, L. M. , Huemer, J. , Carreon, D. M. , Jiang, Y. , Eickhoff, S. B. , & Etkin, A. (2017). Identification of common neural circuit disruptions in cognitive control across psychiatric disorders. The American Journal of Psychiatry, 174, 676–685. 10.1176/appi.ajp.2017.16040400 28320224 PMC5543416

[hbm25772-bib-0035] Menon, V. (2011). Large‐scale brain networks and psychopathology: A unifying triple network model. Trends in Cognitive Sciences, 15, 483–506. 10.1016/j.tics.2011.08.003 21908230

[hbm25772-bib-0036] Miller, K. , Alfaro‐Almagro, F. , Bangerter, N. , Thomas, D. L. , Yacoub, E. , Xu, J. , … Smith, S. M. (2016). Multimodal population brain imaging in the UK biobank prospective epidemiological study. Nature Neuroscience, 19, 1523–1536. 10.1038/nn.4393 27643430 PMC5086094

[hbm25772-bib-0037] Noble, S. , Scheinost, D. , & Constable, R. T. (2019). A decade of test–retest reliability of functional connectivity: A systematic review and meta‐analysis. NeuroImage, 203, 116157. 10.1016/j.neuroimage.2019.116157 31494250 PMC6907736

[hbm25772-bib-0038] Ohi, K. , Ochi, R. , Noda, Y. , Wada, M. , Sugiyama, S. , Nishi, A. , … Nakajima, S. (2021). Polygenic risk scores for major psychiatric and neurodevelopmental disorders contribute to sleep disturbance in childhood: Adolescent brain cognitive development (ABCD) study. Translational Psychiatry, 11, 1–11. 10.1038/s41398-021-01308-8 33771979 PMC7997961

[hbm25772-bib-0039] Sämann, P. G. , Tully, C. , Spoormaker, V. I. , Wetter, T. C. , Holsboer, F. , Wehrle, R. , & Czisch, M. (2010). Increased sleep pressure reduces resting state functional connectivity. Magnetic Resonance Materials in Physics, Biology and Medicine, 23, 375–389. 10.1007/s10334-010-0213-z 20473549

[hbm25772-bib-0040] Scott, N. , Blair, P. S. , Emond, A. M. , Fleming, P. J. , Humphreys, J. S. , Henderson, J. , & Gringras, P. (2013). Sleep patterns in children with ADHD: A population‐based cohort study from birth to 11 years. Journal of Sleep Research, 22, 121–128. 10.1111/j.1365-2869.2012.01054.x 23057438 PMC3786528

[hbm25772-bib-0041] Sha, Z. , Wager, T. D. , Mechelli, A. , & He, Y. (2019). Common dysfunction of large‐scale neurocognitive networks across psychiatric disorders. Biological Psychiatry, 85, 379–388. 10.1016/j.biopsych.2018.11.011 30612699

[hbm25772-bib-0042] Tesler, N. , Gerstenberg, M. , & Huber, R. (2013). Developmental changes in sleep and their relationships to psychiatric illnesses. Current Opinion in Psychiatry, 26, 572–579. 10.1097/YCO.0b013e328365a335 24060918

[hbm25772-bib-0043] Wager, T. D. , Davidson, M. L. , Hughes, B. L. , Lindquist, M. A. , & Ochsner, K. N. (2008). Prefrontal‐subcortical pathways mediating successful emotion regulation. Neuron, 59, 1037–1050. 18817740 10.1016/j.neuron.2008.09.006 PMC2742320

[hbm25772-bib-0044] Wager, T. D. , Waugh, C. E. , Lindquist, M. , Noll, D. C. , Fredrickson, B. L. , & Taylor, S. F. (2009). Brain mediators of cardiovascular responses to social threat: Part I: Reciprocal dorsal and ventral sub‐regions of the medial prefrontal cortex and heart‐rate reactivity. NeuroImage, 47, 821–835. 10.1016/j.neuroimage.2009.05.043 19465137 PMC3275821

[hbm25772-bib-0045] Woo, C.‐W. , Chang, L. J. , Lindquist, M. A. , & Wager, T. D. (2017). Building better biomarkers: Brain models in translational neuroimaging. Nature Neuroscience, 20, 365–377. 10.1038/nn.4478 28230847 PMC5988350

[hbm25772-bib-0046] Xia, C. H. , Ma, Z. , Ciric, R. , Gu, S. , Betzel, R. F. , Kaczkurkin, A. N. , … Satterthwaite, T. D. (2018). Linked dimensions of psychopathology and connectivity in functional brain networks. Nature Communications, 9, 3003. 10.1038/s41467-018-05317-y PMC6070480 30068943

[hbm25772-bib-0047] Yang, F. N. , Bronshteyn, M. , Flowers, S. A. , Dawson, M. , Kumar, P. , Rebeck, G. W. , … Jiang, X. (2021). Low CD4 nadir exacerbates the impacts of APOE ε4 on functional connectivity and memory in adults with HIV. AIDS, 35, 727–736. 10.1097/QAD.0000000000002840 33587445 PMC8318747

[hbm25772-bib-0048] Yu, M. , Linn, K. A. , Cook, P. A. , Phillips, M. L. , McInnis, M. , Fava, M. , … Sheline, Y. I. (2018). Statistical harmonization corrects site effects in functional connectivity measurements from multi‐site fMRI data. Human Brain Mapping, 39, 4213–4227. 10.1002/hbm.24241 29962049 PMC6179920

