# Supplementary material for: Functional connectome mediates the association between sleep disturbance and mental health in preadolescence: A longitudinal mediation study
Source: Hum Brain Mapp. 2022 Jan 18;43(6):2041–50. doi: 10.1002/hbm.25772 (PMC8933321; doi:10.1002/hbm.25772)
Supplement: Supplementary file 1 — Appendix S1: Supporting information. [file HBM-43-2041-s001.docx]

**Supplemental Materials**

**Effect of sex on the association between total sleep disturbance and mental problems**

We performed two additional analyses to test whether sex difference exists in the relationship between total sleep disturbance (TSD) and mental problems (MP). Specifically, linear mixed effect model was used. Model 1: TSD was the dependent variable, MP was the independent variable, and the MP and sex interaction was tested. Model 2: MP was the dependent variable, TSD was the independent variable, the TSD and sex interaction was tested. All models included fixed-effect covariates for age, sex at birth, race (black, white, and others), pubertal status (1-4, assessed by ABCD Youth Pubertal Development Scale and Menstrual Cycle Survey History), average motion during resting scan (mean FD), and random effects for family relatives nested within data collection sites.

The interaction term in neither model was not significant (model 1: *p* = 0.868, model 2: *p* = 0.725), suggesting that sex did not affect the relationship between TSD and MP.


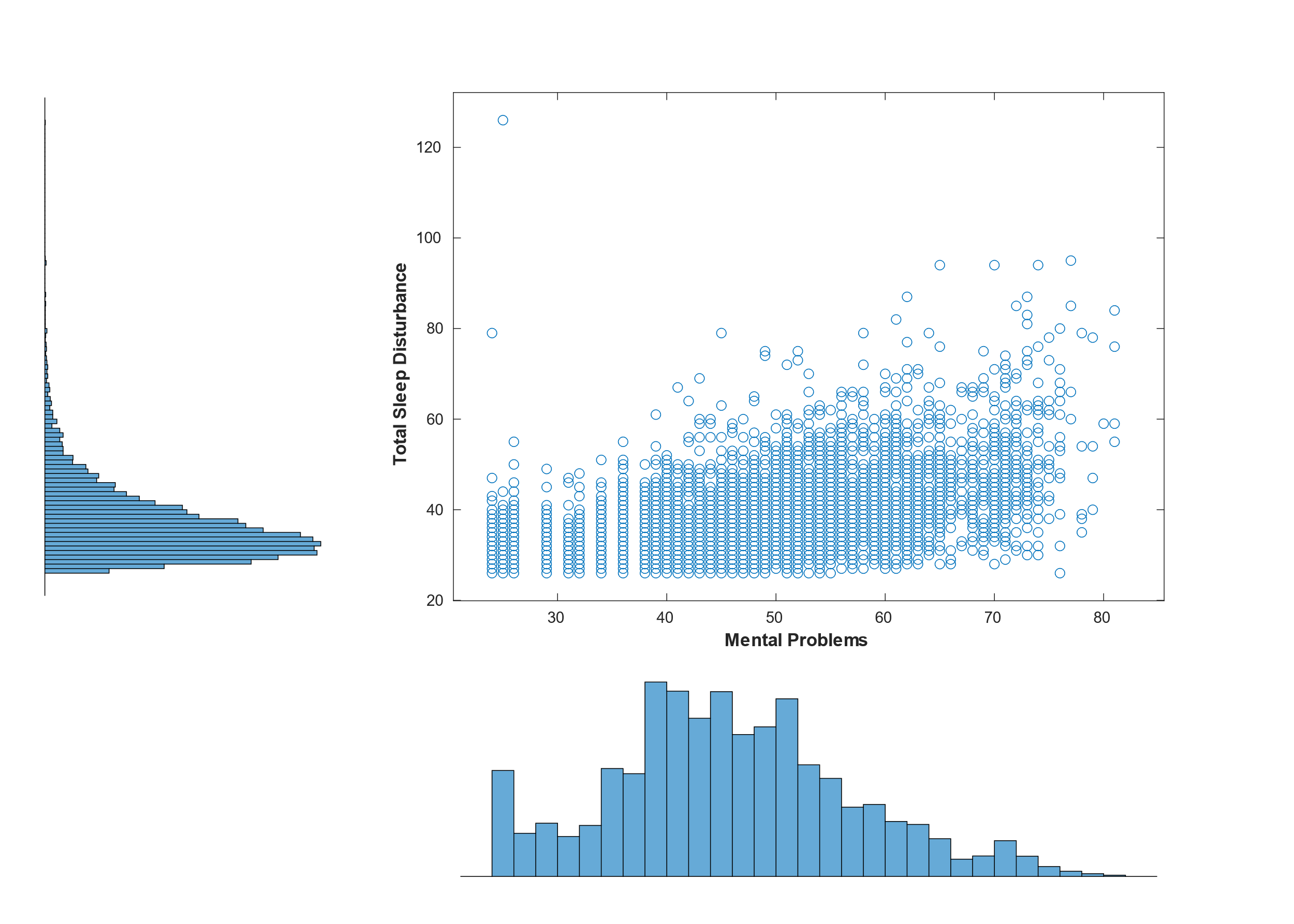


**Figure S1: Correlation between total sleep disturbance and** **mental problems.** TSD and MP were significantly correlated (*r* = 0.568, *p* < 1e-10), after controlling for age, sex, race, pubertal status, effects for family relatives, mean FD, and data collection sites. Left histogram shows the distribution of TSD. Bottom histogram shows the distribution of MP. TSD: total sleep disturbance; MP: mental problems.

**Relationships between cognition, identified network connectivity, total sleep disturbance and mental problems**

We performed a partial correlation analysis between cognition and identified network connectivities (DMN-DAN, DAN-DAN, DMN-DMN) and between cognition and TSD/MP, controlling for age, sex, race, pubertal status, effects for family relatives, and data collection sites. Further mediation analysis was performed to test whether identified network connectivities mediated the effects of TSD or MP on cognition.

Partial correlation analysis showed that DMN-DAN and DAN-DAN were significantly correlated with the Total Cognitive Composite Fully-Corrected T-score (total cognition, *r* = -0.0361 and 0.0231, *p* = 0.0008 and 0.0321, *q* = 0.0008 and 0.301 with 10,000 permutation, FDR corrected *p* = 0.0025 and 0.0483, respectively, see Fig. S2), after controlling for age, sex, race, pubertal status, effects for family relatives, and data collection sites, suggesting that these two network connectivities are important factors that influence general cognitive functions in preadolescence. Total cognition also significantly correlated with total sleep disturbance (r = -0.0287, p = 0.009) and mental problems (r = -0.102, p < 1e-10), after controlling for age, sex, race, pubertal status, effects for family relatives, mean FD, and data collection sites.

Further mediation analysis showed that, DMN-DAN mediated the effect of total sleep disturbance on total cognition (beta of a*b = -0.004, *p* < 0.001), and the effect of mental problems on total cognition (beta of a*b = -0.003, *p* < 0.001), after controlling for age, sex, race, pubertal status, effects for family relatives, and data collection sites. DAN-DAN mediated the effect of total sleep disturbance on total cognition (beta = -0.0014, *p* = 0.0172), and the effect of mental problems on total cognition (beta of a*b = -0.0018, *p* < 0.01), after controlling for the above-mentioned covariates.

When full covariates were taken into accounts, the mediation effects stayed the same. Specifically, DMN-DAN mediated the effect of total sleep disturbance on total cognition (beta of a*b = -0.004, *p* < 0.001), and the effect of mental problems on total cognition (beta of a*b = -0.003, *p* < 0.001), after controlling for age, sex, race, pubertal status, effects for family relatives, data collection sites, average motion during resting scan (mean framewise-displacement, FD), number of fMRI time points remained after preprocessing, household income, parents’ education, and body mass index. DAN-DAN mediated the effect of total sleep disturbance on total cognition (beta = -0.0014, *p* = 0.0171), and the effect of mental problems on total cognition (beta of a*b = -0.0018, *p* < 0.01), after controlling for above-mentioned covariates.


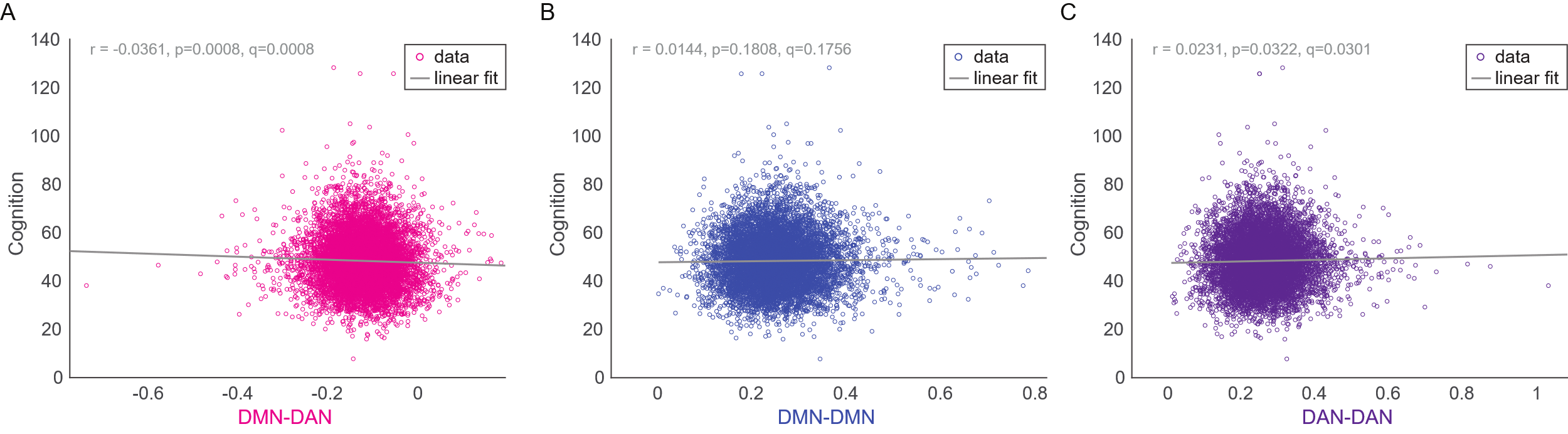


Figure S2. Correlation between network connectivities and total cognition. A. between-network connectivity DMN-DAN significantly correlated with total cognition (*r* = -0.0361, *p* = 0.0008, *q* = 0.0008 with 10,000 permutations). B. correlation between within-network connectivity DMN-DMN and total cognition did not reach statistical significance (*r* = 0.0144, *p* = 0.1808, *q* = 0.1756 with 10,000 permutations). C. within-network connectivity DAN-DAN significantly correlated with total cognition (*r* = 0.0231, *p* = 0.0322, *q* = 0.0301 with 10,000 permutations). DMN, default mode network; DAN, dorsal attention network.


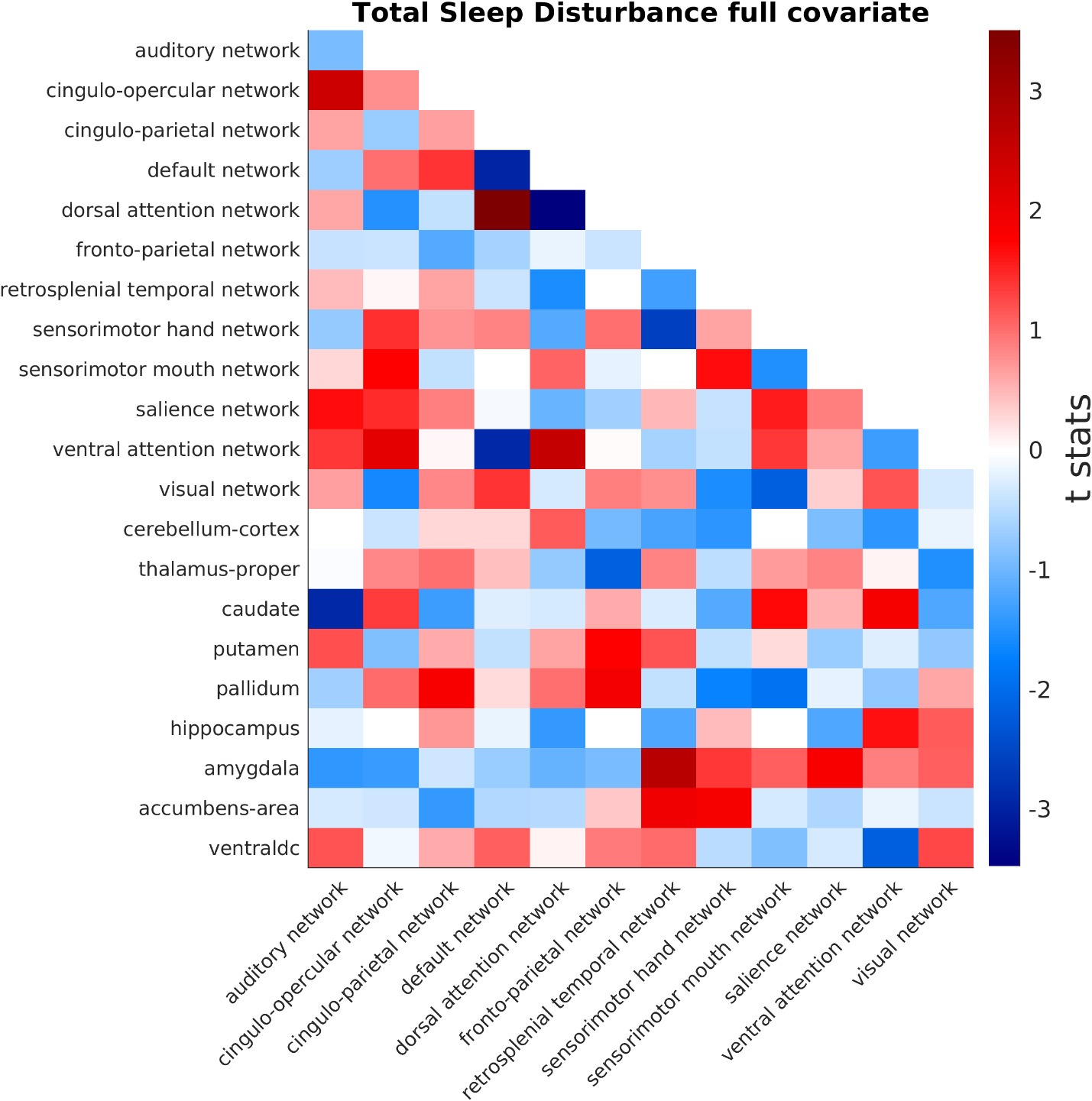


Figure S3. The effect of total sleep disturbance on network connectivity measures. Color represents t-stats. That is, red/blue means positive/negative association between total sleep disturbance and network connectivity measures, respectively. Only unique network connectivity measures were shown in the matrix (i.e., top right was intentionally left blank). Models used here included fixed-effect covariates for age, sex at birth, race (black, white, and others), pubertal status (1-4, assessed by ABCD Youth Pubertal Development Scale and Menstrual Cycle Survey History), average motion during resting scan (mean framewise-displacement, FD), number of fMRI time points remained after preprocessing, household income, parents’ education, body mass index, and random effects for family relatives nested within data collection sites. Ventraldc: ventral Diencephalon.


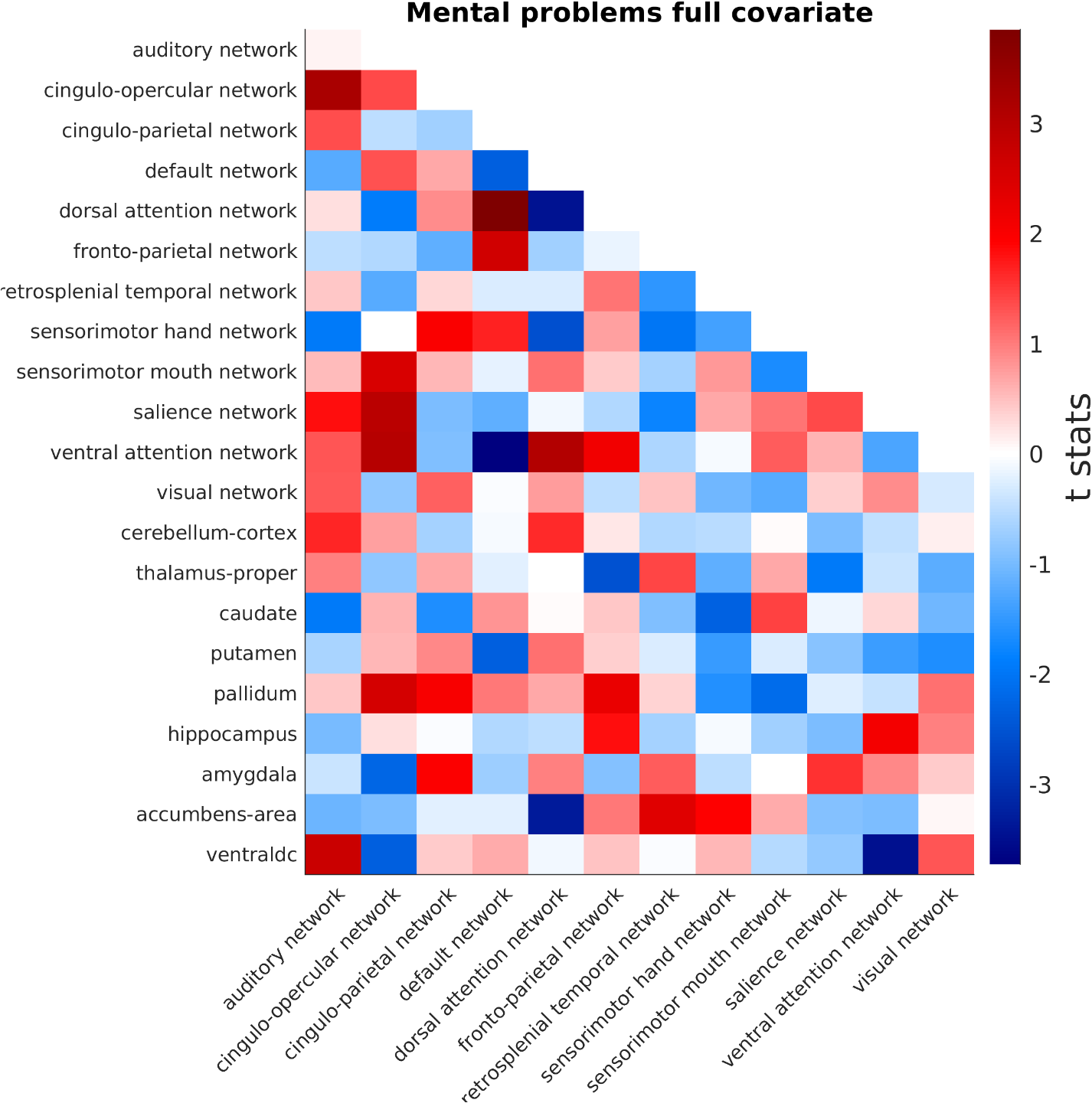


Figure S4. The effect of mental problems on network connectivity measures. Color represents t-stats. That is, red/blue means positive/negative association between mental problems and network connectivity measures, respectively. Only unique network connectivity measures were shown in the matrix (i.e., top right was intentionally left blank). Models used here included fixed-effect covariates for age, sex at birth, race (black, white, and others), pubertal status (1-4, assessed by ABCD Youth Pubertal Development Scale and Menstrual Cycle Survey History), average motion during resting scan (mean framewise-displacement, FD), number of fMRI time points remained after preprocessing, household income, parents’ education, body mass index, and random effects for family relatives nested within data collection sites. Ventraldc: ventral Diencephalon.
